# Bone resorptive activity in symptomatic and asymptomatic apical lesions of endodontic origin

**DOI:** 10.1007/s00784-017-2062-x

**Published:** 2017-03-06

**Authors:** M. Salinas-Muñoz, M. Garrido-Flores, M. Baeza, P. Huamán-Chipana, J. García-Sesnich, R. Bologna, R. Vernal, M. Hernández

**Affiliations:** 1 Laboratory of Periodontal Biology, Faculty of Dentistry, Universidad de Chile, Santiago, Chile; 2 Department of Conservative Dentistry, Faculty of Dentistry, Universidad de Chile, Santiago, Chile; 3 Molecular Pathology Area, School of Dentistry, Universidad de la República UDELAR, Montevideo, Uruguay; 4 Dentistry Unit, Faculty of Health Sciences, Universidad Autónoma de Chile, Santiago, Chile; 5 Department of Oral Pathology and Medicine, Faculty of Dentistry, Universidad de Chile, Av. Sergio Livingstone 943, Independencia, Santiago, Chile

**Keywords:** Symptomatic, Asymptomatic periapical periodontitis, Bone resorption, Biomarkers, TRAP, RANKL, OPG

## Abstract

**Objectives:**

The aim of this study is to assess the levels and diagnostic accuracy of a set of bone resorption biomarkers, including TRAP-5, RANKL, and OPG in symptomatic and asymptomatic apical lesions and controls.

**Materials and methods:**

Apical tissues from symptomatic and asymptomatic apical periodontitis patients and periodontal ligaments from healthy teeth extracted for orthodontic reasons were processed for tissue homogenization and the levels of TRAP-5, RANKL, and OPG were determined by multiplex assay. Marker levels were analyzed by Kruskal Wallis test, and diagnostic accuracy was analyzed with ROC curves.

**Results:**

Higher levels of RANKL, OPG, and RANKL/OPG ratio were determined in both types of apical lesions compared to healthy periodontal ligament, whereas higher TRAP-5 levels were found only in symptomatic apical lesions (*p* < 0.05). OPG, RANKL, and RANKL/OPG ratio showed diagnostic potential to identify apical lesions versus healthy controls (AUC = 0.69, *p* < 0.05); while TRAP-5 showed a potential to discriminate symptomatic versus asymptomatic apical periodontitis (AUC = 0.71, *p* < 0.05) and healthy controls (AUC = 0.83, *p* < 0.05).

**Conclusions:**

Apical lesions showed higher RANKL and OPG levels than healthy tissues. TRAP-5 levels were the highest in symptomatic apical lesions, suggesting that these represent a progressive state, and showed diagnostic potential.

**Clinical relevance:**

Clinically symptomatic apical periodontitis might represent biologically progressive apical lesions based on TRAP5 levels. TRAP5 has diagnostic potential to identify these lesions, representing a candidate prognostic biomarker.

## Introduction

Apical periodontitis originates from host’s immune response to a dominant Gram-negative anaerobic biofilm localized inside the root canal system of the tooth and their respective by-products. Given the inability to eliminate bacteria, the host attempts to localize the infection and prevent further dissemination at the expense of apical tissue breakdown that results in the formation of an osteolytic apical lesion (AL), the hallmark of chronic forms of apical periodontitis [1].

AL is heterogeneous from a clinical point of view depending on its association with clinical symptoms, being either symptomatic apical periodontitis (SAP) or asymptomatic apical periodontitis (AAP) [2]. This clinical variability is expected to depend on the dynamic balance between bacterial consortia and the host’s response [3–5]. Recent studies support that symptomatic apical periodontitis associates with changes in bacterial load and diversity [4], as well as host’s immune response, involving interleukin (IL)-1, IL-6, tumor necrosis factor (TNF)-α, and matrix metalloproteinase (MMP)-9, respectively [6–8]. Although the clinical diagnosis of symptomatic apical periodontitis is straightforward, its biologic significance remains to be known.

Osteoclasts are the final cellular effectors of bone resorption, determining progression versus healing processes in AL. Osteoclast differentiation and activation from its monocytic precursors is regulated in part through the balance between the receptor activator of nuclear factor κB (RANK), its ligand (RANKL), and its decoy receptor, osteoprotegerin (OPG). Accordingly, significantly higher RANKL levels have been reported in AL compared with healthy tissues [9]. RANKL/OPG ratio has also been proposed as an indicator of AL progression [10, 11]. Tartrate-resistant acid phosphatase (TRAP)-5 on the other hand, is an enzyme released along with bone matrix degradation products by active osteoclasts, representing a direct biomarker of osteoclastic activity and bone resorption [12–17].

Higher levels of TRAP-5 are associated with the progression of bone destructive diseases; there is a current need to identify biomarkers for AL progression [18]; however, up to now there are no clinical studies linking TRAP-5 with apical periodontitis [19, 20]. Furthermore, bone resorptive dynamics in symptomatic and asymptomatic states of apical periodontitis remain unknown [7]. We aimed to assess the levels and diagnostic accuracy of a set of bone resorption biomarkers in AL from patients with clinical diagnoses of SAP, AAP, and healthy periodontal ligaments as controls, including TRAP-5, RANKL, and OPG.

## Materials and methods

### Materials

Tris-HCl pH 7.5, NaCl, CaCl_2_, and Triton X-100 were purchased from Sigma-Aldrich (St Louis, MO, USA) for homogenization buffer preparation. EDTA-free proteinase inhibitor cocktail was purchased from Roche Diagnostics GmbH (Mannheim, Germany). A Milliplex MAP multiplex assay panel (human cancer/metastasis biomarker magnetic bead) and human bone RANKL single-plex panel was obtained from Millipore, Merck KGaA (Darmstadt, Germany).

### Methods

Patients who consulted at the Clinic of Oral Surgery, School of Dentistry, University of Chile, Santiago, Chile, were enrolled if they had a clinical diagnosis of either SAP or AAP in the presence of an apical lesion detected by apical radiography (>2 mm diameter) caused by dental caries in teeth with a clinical diagnosis of nonvital pulp, according to previously defined criteria [3]. Periodontal ligaments obtained from healthy premolars extracted for orthodontic reasons were used as controls as previously described [21, 22]. Exclusion criteria included systemic illness or previous antibiotics or nonsteroidal anti-inflammatory treatment during a 6-months period before the study [23]. All procedures were performed in accordance with the ethical standards of the institutional research and ethics committee and with the Helsinki declaration. The investigation protocol was clearly explained to all the participants of this study. Each participant signed an informed consent or corresponding forms for their legal guardians in case of underage patients after the risks and benefits of participation were described. A total of 52 apical lesions from patients with SAP (*n* = 17) and AAP (*n* = 35), and periodontal ligament samples from healthy volunteers (*n* = 24) were obtained. After tooth extraction, apical lesions and healthy periodontal ligaments were extracted by surgical separation from the tooth surface with curettes and then stored at −20 °C until processed for tissue homogenization and multiplex assay.

#### Tissue homogenates and multiplex assay

After thawing, tissue samples from AL (*n* = 52) and control periodontal ligaments (*n* = 24) were weighted. Protein extracts were obtained by manual homogenization in 50 mM Tris-HCl pH 7.5, 0.2 mM NaCl, 5 mM CaCl_2_, and 0.01% Triton X-100 (Sigma-Aldrich, St Louis, MO) buffer adding EDTA-free proteinase inhibitor cocktail (Roche Diagnostics GmbH, Mannheim, Germany) in a constant ratio of 10:1 μL of buffer per milligram of weighted tissue; centrifuged at 10,000×*g* for 6 min at 4 °C and stored at −80 °C until further analysis with Milliplex MAP multiplex assays (human cancer/metastasis biomarker magnetic bead and human bone RANKL single-plex panels, Millipore, Merck KGaA, Darmstadt, Germany), according to the manufacturer’s instructions. Data was read through a Luminex platform (Magpix, Millipore, St Charles, MO, USA), and analyzed with the MILLIPLEX AnalystR software (ViageneTech, Carlisle, MA, USA).

#### Statistical analyses

Comparisons of TRAP-5, RANKL, and OPG levels between SAP and AAP and controls were analyzed with ANOVA or Kruskal Wallis test using STATA V.11 (StataCorp, College Station, TX, USA), according to data distribution. The evaluation of the diagnostic accuracy of the biomarkers was performed through the construction of ROC curves using SPSS19 software (IBM® Company, Armork, NY, USA) by calculating the area under the curve (AUC). The optimal cut-off points to estimate the highest sensitivity and specificity altogether were assessed by Youden’s index. A *p* value <0.05 was considered statistically significant.

## Results

**Fig. 1:**
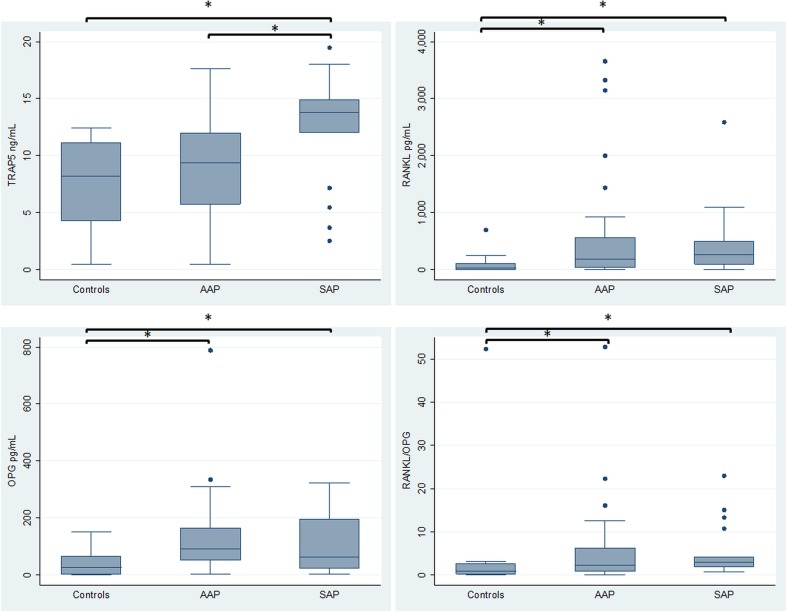
Bone resorption biomarkers levels in healthy controls, AAP and SAP. Control: healthy periodontal ligaments; *AAP* asymptomatic apical periodontitis; *SAP* symptomatic apical periodontitis; *TRAP5* tartrate-resistant acid phosphatase-5; *RANKL* receptor activator for nuclear factor κB ligand; *OPG* osteoprotegerin; *RANKL/OPG* ratio between RANKL and OPG. *Bars* and *asterisks* represent significant (*p* < 0.05) pairwise comparisons (ANOVA and Tukey test for TRAP-5 and Kruskal Wallis test for RANKL, OPG, and RANKL/OPG ratio)

Controls, asymptomatic apical periodontitis and symptomatic apical periodontitis groups had a mean age of 13.7, 50.1, and 46.5 years and 7, 12, and 11 were women, respectively. No smokers were reported in the controls, whereas there were six smokers in each apical periodontitis group. Regarding biomarker levels (Fig. 1), significantly higher levels of TRAP-5 were observed in SAP in comparison to AAP and healthy controls (*p* < 0.05), and with no differences between the latter two groups. The levels of RANKL, OPG, and the RANKL/OPG ratio were significantly higher in AAP and SAP groups compared with healthy controls (*p* < 0.05), and no differences were found between both apical periodontitis groups.

**Fig. 2:**
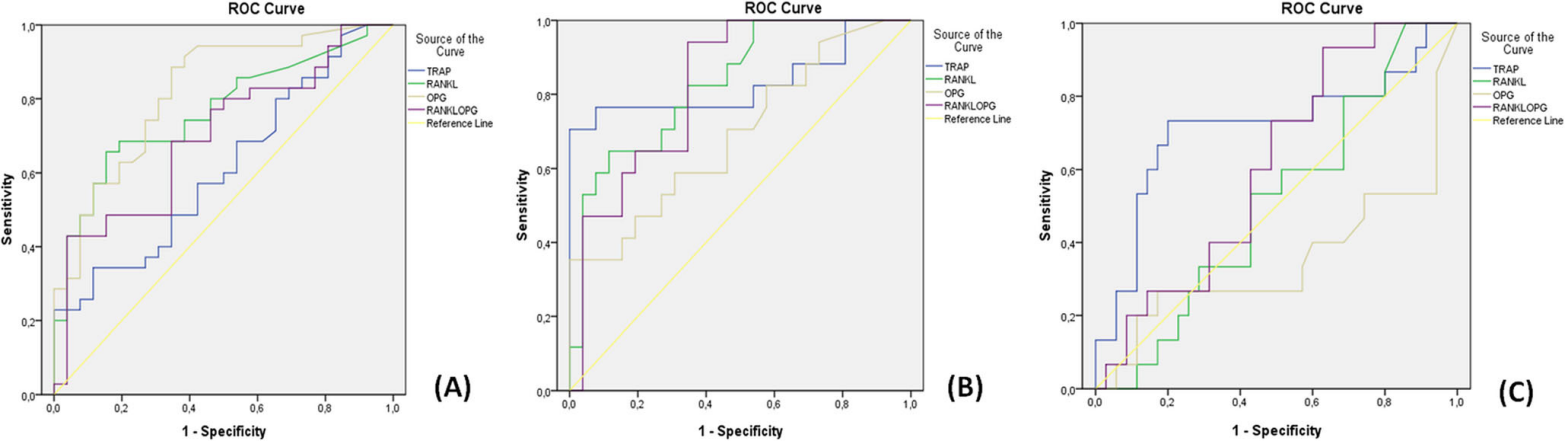
ROC curves for the diagnosis of AAP versus controls (**a**), SAP versus controls (**b**), and AAP versus SAP (**c**). *AAP* asymptomatic apical periodontitis; *SAP* symptomatic apical periodontitis; *y* axis: sensitivity, *x* axis: 1-specificity. Lines: *blue* tartrate-resistant acid phosphatase-5 (TRAP), *green* receptor activator for nuclear factor κB ligand (RANKL), *gray* osteoprotegerin (OPG), *purple* ratio between RANKL, and OPG, *yellow* reference line

The diagnostic performance of biomarkers is illustrated with ROC curves (Fig. 2a, b, c) and their respective values are presented in Table 1a, b, c. RANKL, OPG, and RANKL/OPG demonstrated statistically significant (*p* < 0.05) diagnostic accuracy to identify AAP versus healthy controls, where the highest performance corresponded to RANKL (area under the curve =0.77, 95% CI 0.65–0.89), [Fig. 2a and Table 1a ]. Optimal cut-off points were obtained for each marker by using Youden’s index. OPG showed the best performance, with a sensitivity of 80% and a specificity of 69% at a cut-off point of 50 pg/mL, followed by RANKL and RANKL/OPG ratio.

**Table 1:** Accuracy of biological markers for the diagnosis of AA versus controls (a), SAP versus controls (b) and AAP versus SAP (c)

Biomarker	Cut-off	Sensitivity	Specificity	Accuracy (AUC, CI)	*p*
(a)					
TRAP-5	8.45 ng/mL	0.57	0.58	0.61 (0.47–0.75)	*0.143*
RANKL	122.00 pg/mL	0.66	0.85	0.77 (0.65–0.89)	*<0.0001*
OPG	50.0 pg/mL	0.80	0.69	0.82 (0.71–0.93)	*<0.0001*
RANKL/OPG	1.05	0.69	0.66	0.70 (0.57–0.83)	*0.009*
(b)					
TRAP-5	12.48 ng/mL	0.71	1.00	0.83 (0.68–0.98)	*<0.0001*
RANKL	90.64 pg/mL	0.77	0.69	0.83 (0.71–0.95)	*<0.0001*
OPG	51.50 pg/mL	0.59	0.69	0.69 (0.53–0.86)	*0.035*
RANKL/OPG	0.99	0.94	0.65	0.82 (0.70–0.95)	*<0.0001*
(c)					
TRAP-5	12.54 ng/mL	0.73	0.80	0.71 (0.54–0.89)	*0.017*
RANKL	170.5 pg/mL	0.53	0.57	0.51 (0.34–0.68)	0.92
OPG	70.50 pg/mL	0.40	0.60	0.35 (0.16–0.54)	0.10
RANKL/OPG	2.40	0.73	0.51	0.61 (0.45–0.77)	0.22

*AAP* asymptomatic apical periodontitis; *SAP* symptomatic apical periodontitis; *AUC* area under the curve; *CI* confidence interval of 95%; *TRAP5* tartrate-resistant acid phosphatase-5; *RANKL* receptor activator for nuclear factor κB ligand; *OPG* osteoprotegerin; *RANKL/OPG* ratio between RANKL and OPG

*p*<0.05

In the construction of ROC curves for the diagnosis of SAP versus healthy controls [Fig. 2b and Table 1b], TRAP-5 (AUC = 0.83, 95% CI 0.68–0.98), and RANKL (AUC = 0.83, 95% CI 0.71–0.95) were the markers with the highest accuracy, followed by RANKL/OPG ratio and OPG (*p* < 0.05). According to Youden’s index, TRAP-5 showed a sensitivity of 71%, a specificity of 100%, at a cut-off point of 12.48 ng/mL, followed by RANKL with a sensitivity of 77% and a specificity of 69% at a cut-off point of 90.64 pg/mL.

TRAP-5 (AUC = 0.71, 95% CI 0.54–0.89) was the only biomarker that showed potential to discriminate between SAP and AAP [Fig. 2c) and Table 1c, *p* < 0.05]. TRAP-5 showed a sensitivity of 73% and a specificity of 80% at a cut-off point of 12.54 ng/mL.

## Discussion

AL results from an imbalanced osteoclastic activity induced by the immuno-inflammatory response to endodontic bacterial infection [24]. Clinically symptomatic ALs have been suggested to represent an immunologically active stage of the disease [7, 8], but up to now, bone resorptive activity had not been established. In the present study, we assessed the levels of bone resorption markers in symptomatic and asymptomatic AL and healthy periodontal ligament as controls and found statistically significant higher levels of RANKL and OPG in both types of apical lesions compared to healthy periodontal ligament, while higher TRAP-5 levels were found only in symptomatic AL, suggesting that the later represent progressive lesions. TRAP-5 had diagnostic potential for SAP, representing a potentially useful candidate biomarker for apical periodontitis progression.

Bone resorption is a multistep process where RANK/RANKL/OPG pathway and TRAP-5 play key roles. Through binding RANK, RANKL is among the key osteolytic cytokines that promote osteoclast maturation and activation. OPG on the other hand, is a decoy receptor that prevents the coupling of RANK to RANKL [10]. Unlike the RANKL/RANK axis, which might either result or not in osteoclast differentiation depending on the OPG levels, TRAP-5 is an enzyme released along with bone matrix degradation products by active osteoclasts representing a direct biomarker of osteoclastic activity and bone resorption [12–14, 19, 20].

In the present study the levels of RANKL, OPG and RANKL/OPG ratio were significantly higher in symptomatic and asymptomatic apical lesions versus healthy controls, whereas no significant differences were found between both lesion types. Previous reports associate high levels of RANKL and OPG with apical lesions [10, 17, 25]. In line with our results, a recent report showed no differences in RANKL and OPG levels between asymptomatic and symptomatic apical lesions, whereas a significantly higher number of gram-negative bacteria along with a positive correlation with OPG were found in the later [26]. Whereas the participants showed an homogeneous distribution by gender, an intrinsic methodologic drawback for including healthy periodontal ligament as controls is the age difference among healthy and AP groups, although it represents the closest histophysiologic counterpart for ALs in humans [22]. Accordingly, higher mRNA expression levels were found for RANKL and OPG in apical granulomas versus periodontal ligaments [10]. Recently, RANKL was identified at higher levels in apical exudates from asymptomatic apical periodontitis compared to irreversible pulpitis, whereas OPG levels remained mostly undetectable [27]. So far, there are no previous studies that associate the RANKL/OPG levels with the clinical status in AP. The increased levels of RANKL, OPG, and RANKL/OPG ratio in ALs suggest that these lesions display an imbalance towards osteoclast differentiation, though it might not necessarily result in higher osteolysis [10, 27]. Based on our results, these markers did not differentiate between symptomatic and asymptomatic apical lesions.

TRAP-5 levels were significantly higher in symptomatic apical lesions compared with asymptomatic lesions and healthy controls, while no differences were found between asymptomatic lesions and controls. TRAP-5 has been proposed to be the most reliable marker of bone resorptive activity. Clinical studies, as well as animal and in vitro experimental models, demonstrate that elevated levels of TRAP-5 can be more specifically explained by an increase in the activity of osteoclasts [12–15, 28]. Accordingly, studies conducted in animal models demonstrate that bone resorption rate is significantly higher during the acute stage of apical periodontitis, compared to its chronic phase, where it becomes slower to finally achieve stabilization [29].

Bone resorption and RANKL production by several cell types is synergistically induced by IL-1, IL-6, and TNF-α. In line with our results, clinical studies report significantly higher levels of these cytokines in apical lesions versus pulp tissue controls and symptomatic versus asymptomatic apical lesions [7, 8, 23]. Despite the RANKL/OPG ratio has been proposed as an indicator of progression or stability of asymptomatic lesions [10], osteolytic cytokines are redundant. TNF-α and IL-1 can also stimulate osteoclast differentiation via RANKL/RANK-independent mechanisms [30, 31], implicating that osteoclastogenesis is only partially accounted by the RANKL/RANK axis. Altogether, these antecedents suggest that inflammation enhances osteoclast differentiation in periapical lesions via RANKL/OPG, but only symptomatic lesions might truly represent an active stage, as reflected by concomitantly elevated RANKL/OPG ratio and TRAP-5 levels. Based on previous reports, it is possible that activation pathways other than RANKL might be induced in symptomatic lesions, resulting in exacerbated bone resorption. The detection of RANKL, OPG, and TRAP at lower levels in healthy periodontal ligament on the other hand might reflect bone homeostasis.

The diagnostic accuracy of OPG, RANKL, and RANKL/OPG ratio showed potential to identify apical lesions versus healthy controls, but failed to discriminate between AAP and SAP. Thus, OPG and RANKL could be useful in the diagnosis of apical periodontitis; however, they do not differentiate between its clinical forms. TRAP-5 on the other hand proved to have the highest diagnostic accuracy to discriminate SAP from AAP and healthy controls, presenting potential as a marker for progressive lesions. Accordingly, TRAP levels also demonstrated diagnostic precision to identify marginal chronic periodontitis in a recent study [15]. Currently, apical diagnosis is based solely on clinical and radiological parameters, but they fail to predict treatment outcome in the short term. Thus, TRAP-5 screening in a noninvasive approach might have potential as biomarker for progressive AL to aid clinical decisions in endodontic and restorative procedures in oral fluid samples, such as gingival crevicular fluid, saliva, or in apical exudates during endodontic treatment [15, 32]. In fact, TRAP levels have already shown a good performance to identify chronic periodontitis in gingival crevicular fluid [15]. Future prospective studies are needed to assess its usefulness as a predictive marker.

## Conclusions

Apical lesions showed higher RANKL and OPG levels than healthy tissues and TRAP-5 levels were the highest in symptomatic apical lesions, suggesting that the later represents a progressive disease state that can be identified by TRAP-5.
